# Sleep stage classification using instantaneous heart and breathing rates with a TCN-BiGRU-SE model

**DOI:** 10.3389/fbioe.2026.1863093

**Published:** 2026-08-10

**Authors:** Yi Wang, Jiayong Yan, Chaowen Li, Hui Yang

**Affiliations:** 1 School of Health Science and Engineering, University of Shanghai for Science and Technology, Shanghai, China; 2 College of Medical Instruments, Shanghai University of Medicine & Health Sciences, Shanghai, China

**Keywords:** cardiorespiratory signals, deep learning, multi-modal learning, respiration signals, sleep staging, wearable sleep monitoring

## Abstract

**Background:**

Sleep staging is a fundamental task in clinical sleep medicine and wearable health monitoring. Traditional sleep staging methods primarily rely on polysomnography which provides high accuracy but involves complex instrumentation and high cost, limiting its application in home-based monitoring and large-scale screening.

**Methods:**

To enable a more convenient assessment of sleep structure, this study proposes a multi-modal deep learning approach based on instantaneous heart rate (IHR) and instantaneous breathing rate (IBR). In this study, a TCN-BiGRU-SE network is developed, where the temporal convolutional network (TCN) extracts multi-scale temporal features, the bidirectional gated recurrent unit (BiGRU) captures long-term dependencies, and the squeeze-and-excitation (SE) module enhances feature representation via channel-wise attention. The proposed method is evaluated on two public datasets, Sleep Heart Health Study and MESA, for four-class sleep staging (Wake, rapid eye movement, Light, and Deep).

**Results:**

The proposed method achieves an overall accuracy of 82.19% with a Kappa coefficient of 0.74 on the SHHS dataset, and 80.44% accuracy with a Kappa of 0.70 on the MESA dataset. It outperforms traditional HRV/RRV-based approaches and single-modality models, while maintaining stable performance across datasets.

**Conclusion:**

These results demonstrate that the proposed multi-modal framework provides an effective and scalable solution for sleep staging using cardiorespiratory signals, with good cross-dataset generalization capability and potential for practical wearable applications.

## 1 Introduction

Sleep is a fundamental physiological process essential for maintaining physiological homeostasis and mental health. Its quality is closely associated with cognitive performance, emotional regulation, and cardiovascular function. In recent years, with the accelerating pace of modern lifestyles and increasing work-related stress, overall sleep quality has declined, and sleep disorders have become a major public health concern.

In clinical sleep research, polysomnography (PSG) is regarded as the gold standard for sleep staging, as it simultaneously records multiple physiological signals, including electroencephalogram (EEG), electrooculography (EOG), electromyography (EMG), electrocardiogram (ECG), and respiratory signals, thereby providing comprehensive information for sleep structure assessment (Pandi-Perumal et al., 2014). Early sleep staging was commonly based on the Rechtschaffen and Kales (R&K) standard (Rechtschaffen and Kales, 1968), which categorized sleep into wakefulness (Wake), rapid eye movement (REM), and non-rapid eye movement (NREM) stages. This framework was later refined by the American Academy of Sleep Medicine (AASM), resulting in the widely adopted five-stage classification system (Berry et al., 2012).

With the rapid development of wearable technologies, increasing attention has been directed toward automatic sleep staging using simplified physiological signals. Among these, ECG and respiratory signals are particularly attractive due to their accessibility and strong physiological relevance to sleep regulation. Both heart rate variability (HRV) and respiratory dynamics exhibit distinct patterns across sleep stages, making cardiorespiratory signals a promising alternative for sleep staging. In addition to ECG- and respiration-derived features, respiratory audio signals have also been explored for sleep-related assessment; for example, snoring-sound analysis has been used to classify excitation locations in the upper airway, suggesting that acoustic respiratory information may reflect sleep-related breathing characteristics (Sun et al., 2021).

Earlier cardiorespiratory sleep-staging studies mainly relied on traditional machine learning methods with handcrafted features. In these approaches, HRV and respiratory rate variability (RRV) features were extracted and combined with classifiers such as hidden Markov models, support vector machines, and random forests. For instance, Xiao et al. (2013) utilized HRV features with a random forest classifier and reported strong performance under subject-specific settings, although performance degraded under inter-subject conditions. Ebrahimi et al. (2013); Ebrahimi et al. (2015) further investigated time-domain, frequency-domain, and nonlinear features derived from ECG and respiratory signals, demonstrating the feasibility of cardiorespiratory information for sleep staging. However, these conventional methods remain highly dependent on handcrafted feature design and may be insufficient to capture complex temporal dynamics across subjects and datasets.

Despite these advances, traditional machine learning methods remain heavily dependent on handcrafted features, which are often insufficient to capture the complex temporal dynamics of physiological signals, thereby limiting their generalization capability.

In contrast, deep learning methods enable end-to-end feature learning and have demonstrated strong potential in automatic sleep staging using physiological signals. For example, deep neural networks have been applied to instantaneous heart rate-based sleep staging (Sridhar et al., 2020), while cardiorespiratory-signal-based models have shown promising performance for sleep-stage estimation using heart rate and respiratory information (Bakker et al., 2021). More recently, attention-based and multimodal approaches have been introduced to further enhance temporal representation learning. Luo et al. (2023) proposed a hierarchical attention-based method using movement and cardiopulmonary signals, demonstrating the benefit of attention mechanisms for long-sequence physiological modeling.

Transformer-based architectures have also recently been introduced into sleep staging. For instance, START employed an attention-based cross-modal learning Transformer to enhance multimodal representation learning for automatic sleep staging (Sun et al., 2023), while SleepLog adopted a local-global deep fusion strategy to capture both local temporal features and global contextual information in sleep-stage sequences (Sun et al., 2026). FlexSleepTransformer further explored flexible input-channel configurations for Transformer-based sleep staging across different datasets (Guo et al., 2024). In addition, recent foundation-model studies, such as SleepFM, have demonstrated the potential of large-scale multimodal sleep representation learning from PSG recordings for downstream sleep-related prediction tasks (Thapa et al., 2026).

Nevertheless, several challenges remain. Although previous studies have explored various physiological, acoustic, and multimodal signals, many methods still rely on single-modality inputs, predefined features, or relatively complex sensing settings. In addition, the intrinsic coupling between cardiac and respiratory dynamics has not been fully exploited. Effectively capturing both short-term and long-term temporal dependencies in IHR and IBR signals therefore remains a critical challenge for improving low-burden sleep staging performance.

To address these limitations, this study proposes a multi-modal sleep staging framework based on ECG and respiratory signals. Specifically, instantaneous heart rate (IHR) and instantaneous breathing rate (IBR) are extracted to characterize cardiorespiratory dynamics. A TCN-BiGRU-SE network is designed to capture multi-scale temporal features and long-term dependencies while enhancing feature representation through channel-wise attention. The proposed method aims to improve the modeling of cardiorespiratory coupling and achieve robust sleep staging performance across different datasets.

Although TCN, BiGRU, and SE modules have been widely used in deep learning, the novelty of this study lies in their task-specific integration for sleep staging using instantaneous heart rate (IHR) and instantaneous breathing rate (IBR). Compared with conventional PSG-based approaches or methods using a single temporal or attention mechanism, the proposed TCN-BiGRU-SE model jointly captures multi-scale temporal patterns, bidirectional sleep-stage dependencies, and channel-wise feature importance from IHR and IBR signals. Specifically, the TCN branches extract local and multi-scale temporal representations, the BiGRU layers model contextual dependencies across consecutive sleep epochs, and the SE modules adaptively recalibrate informative features in both modality-specific and fused representations. This integrated design enables the model to better exploit subtle physiological variations in IHR and IBR for low-burden sleep staging.

## 2 Materials and methods

### 2.1 Dataset

The data used in this study were obtained from two publicly available datasets: the Multi-Ethnic Study of Atherosclerosis (MESA) dataset (Chen et al., 2015) and the Sleep Heart Health Study (SHHS) dataset (Quan et al., 1997), both of which include polysomnography (PSG) recordings. The SHHS dataset consists of two subsets, SHHS1 and SHHS2. From both datasets, electrocardiogram (ECG) and respiratory (Resp) signals were extracted for subsequent analysis.

In the MESA dataset, the ECG signal was sampled at 250 Hz and the respiratory signal at 32 Hz. In the SHHS dataset, the ECG sampling rate was 125 Hz in SHHS1 and 250 Hz or 256 Hz in SHHS2, while the respiratory signal was sampled at 10 Hz. These datasets provide long-term overnight recordings with heterogeneous sampling configurations, which enable evaluation under realistic conditions.

Sleep stages in both datasets were manually annotated by experts according to the R&K standard (Rechtschaffen and Kales, 1968), including six stages: Wake, REM, S1, S2, S3, and S4. For consistency with common classification settings, the sleep stages were re-labeled into four categories in this study. Specifically, S1 and S2 were merged as light sleep (Light), and S3 and S4 were merged as deep sleep (Deep), resulting in four classes: Wake, REM, Light, and Deep. The demographic characteristics and sleep-stage distributions of the two datasets are summarized in Table 1.

**TABLE 1:** Demographic characteristics and sleep-stage distributions of the SHHS and MESA datasets.

Characteristic	SHHS	MESA
Age, years	64.49 ± 11.34	69.17 ± 9.04
Male/Female, %	47.17/52.83	46.14/53.86
Wake, n (%)	2,649,500 (31.76%)	1,056,081 (42.60%)
REM, n (%)	1,128,529 (13.53%)	259,259 (10.46%)
Light, n (%)	3,581,479 (42.93%)	1,018,870 (41.09%)
Deep, n (%)	983,391 (11.79%)	145,140 (5.85%)

### 2.2 R-peak detection

**FIGURE 1:**
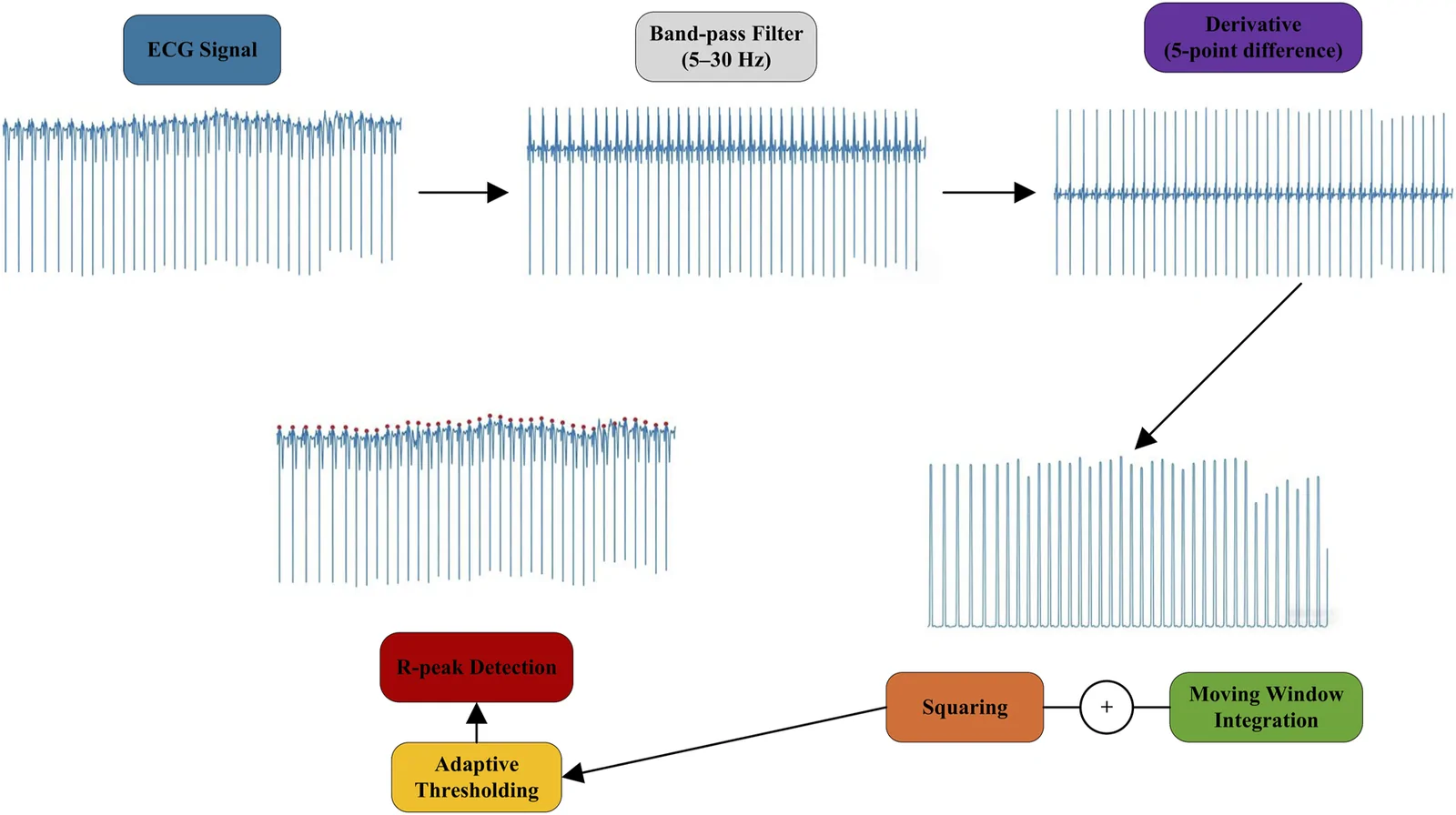
Flowchart of the Pan–Tompkins algorithm for R-peak detection.

ECG and Resp signals were used to derive IHR and IBR, respectively. Specifically, R peaks were detected from the ECG signal to compute RR intervals, while respiratory peaks were identified from the Resp signal to estimate breathing cycles. R-peak detection was performed using the Pan-Tompkins algorithm (Pan and Tompkins, 1985), a widely adopted method for real-time QRS detection in ECG signals. The main processing pipeline is illustrated in Figure 1.

1. The raw ECG signal was filtered using a fourth-order band-pass filter (5–30 Hz) to attenuate baseline drift and high-frequency noise components.The transfer function of the filter is expressed as follows Equation 1:

$$H\left(s\right)=\frac{\omega^{2}}{s^{2}+\frac{\omega }{Q}s+\omega^{2}}$$ (1)

2. Use the five-point difference method to calculate the derivative. This method can effectively estimate the rate of change of the signal (Equation 2):

$$\frac{\text{dECG}}{dt}\approx \frac{‐1\cdot \text{ECG}\left(t‐2\right)‐2\cdot \text{ECG}\left(t‐1\right)+2\cdot \text{ECG}\left(t+1\right)+1\cdot \text{ECG}\left(t+2\right)}{4\Delta t}$$ (2)

In the formula: Δt is the sampling interval.

The differentiated signal was further processed using squaring and moving-window integration to enhance QRS complexes and suppress noise (Equation 3):

$$I\left(t\right)=\frac{1}{N}\sum_{j=0}^{N‐1}{\left(\text{dECG}\left(t‐j\right)\right)}^{2}$$ (3)

4. Adaptive thresholding was subsequently applied for R-peak detection. The thresholds were dynamically adjusted according to the signal characteristics, including a peak-based threshold $T_{\text{peak}}$ and an integrated signal threshold T_integrated_ (Equations 4, 5):

$$T_{\text{peak}}=\text{SPKF}+\alpha \cdot \left(SPKF‐NPKF\right)$$ (4)

$$T_{\text{integrated}}=\text{NPKI}+\beta \cdot \left(SPKI‐NPKI\right)$$ (5)

The adaptive thresholding mechanism maintains dynamic estimates of signal and noise levels for both the filtered and integrated signals. Specifically, the signal peaks (SPKF, SPKI) and noise peaks (NPKF, NPKI) are updated iteratively, with initial values derived from the first 2 s of the corresponding signals. The detection thresholds are then defined as a weighted combination of noise and signal levels, where the weighting factors α and β are set to 0.25.

### 2.3 Respiratory peak detection

Respiratory signals were used to detect breathing peaks and to derive respiratory cycles for IBR calculation. Due to the presence of baseline drift, motion artifacts, and high-frequency noise, robust peak detection is required. In this study, a combination of band-pass filtering and local peak detection was employed.

**FIGURE 2:**
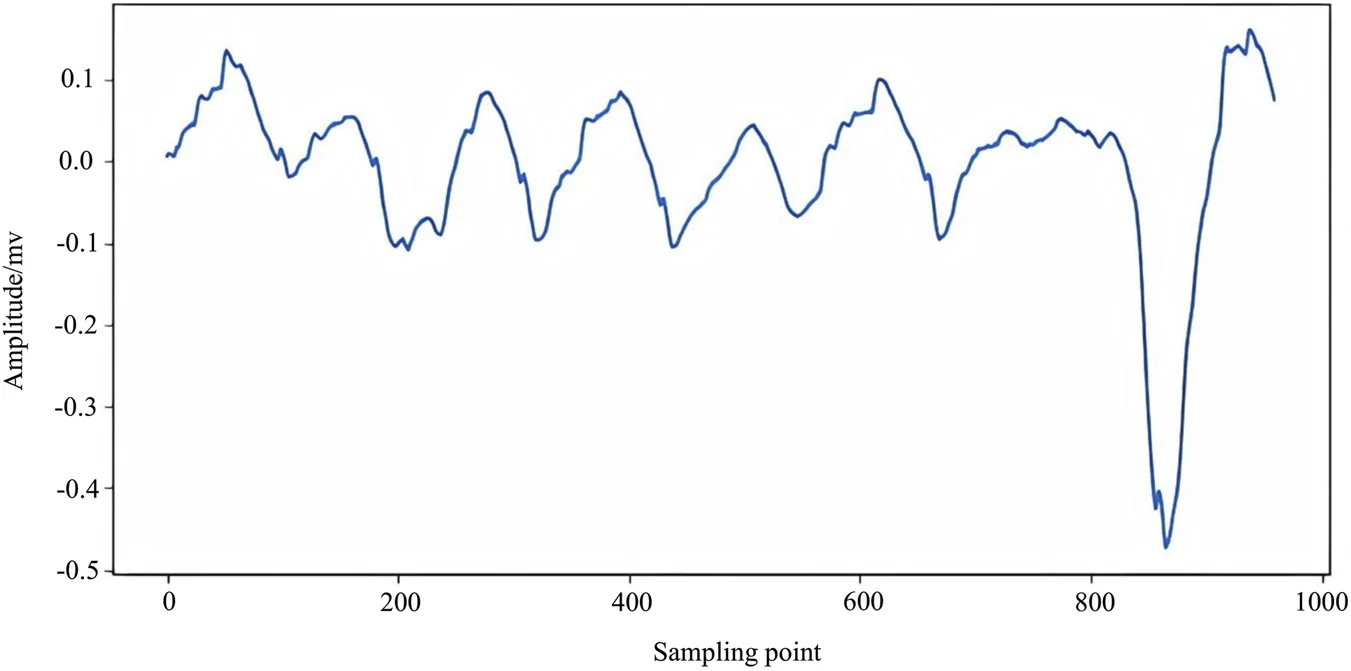
Raw respiratory signal.

**FIGURE 3:**
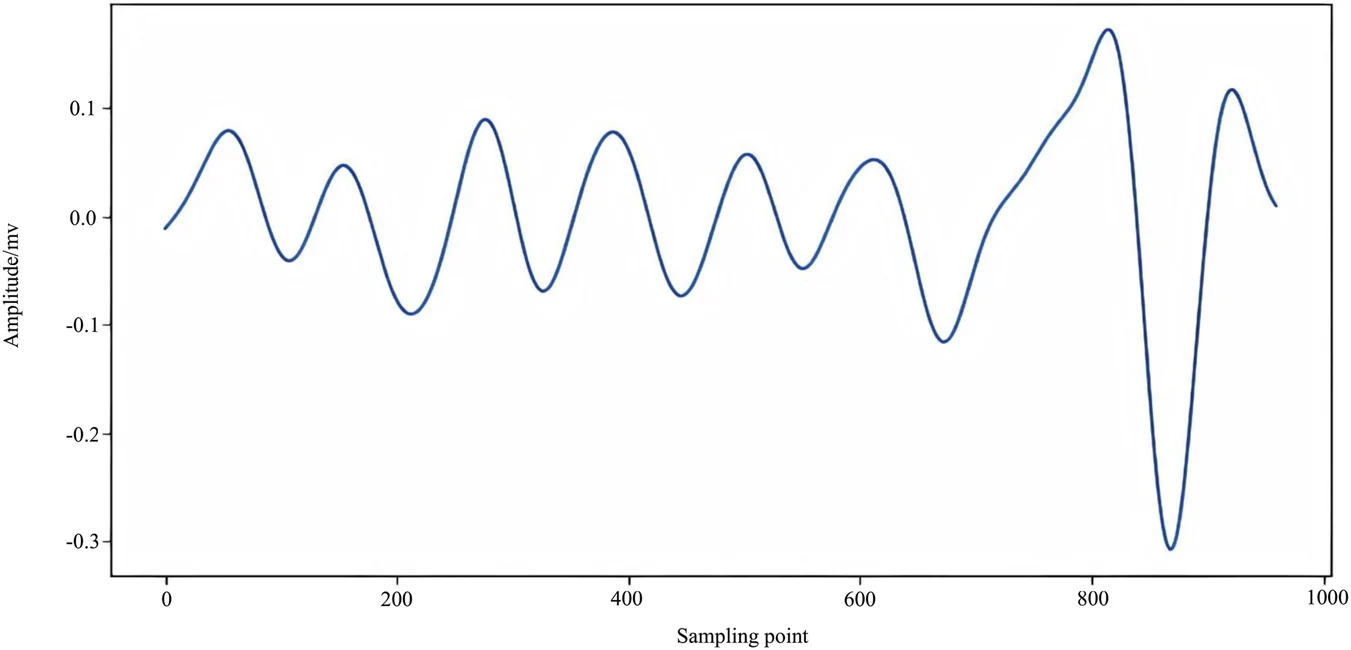
Filtered respiratory signal.

First, the raw respiratory signal was processed using a third-order band-pass filter with a frequency range of 0.1–0.5 Hz. This frequency band corresponds to the normal physiological breathing range and effectively suppresses baseline drift and non-physiological high-frequency noise. The raw and filtered respiratory signals are shown in Figures 2, 3, respectively.

Next, local peaks were detected from the filtered signal using the find_peaks function, which identifies candidate peaks based on comparisons with neighboring samples (Equation 6):

$$x\left(t\right)>x\left(t‐1\right),x\left(t\right)>x\left(t+1\right)$$ (6)

To reduce false detection caused by noise fluctuations and physiologically implausible rapid breathing cycles, two constraints were applied (Equation 7):

1. a minimum peak height of 0 was used to exclude minor residual fluctuations around the baseline;
2. a minimum inter-peak distance was set to 1.7 s.

$$x\left(t\right)-x\left(t‐1\right)>1.7\;s$$ (7)

The distance constraint corresponds to a maximum detectable breathing rate of approximately 35 breaths per minute, which is consistent with typical physiological ranges during sleep.

**FIGURE 4:**
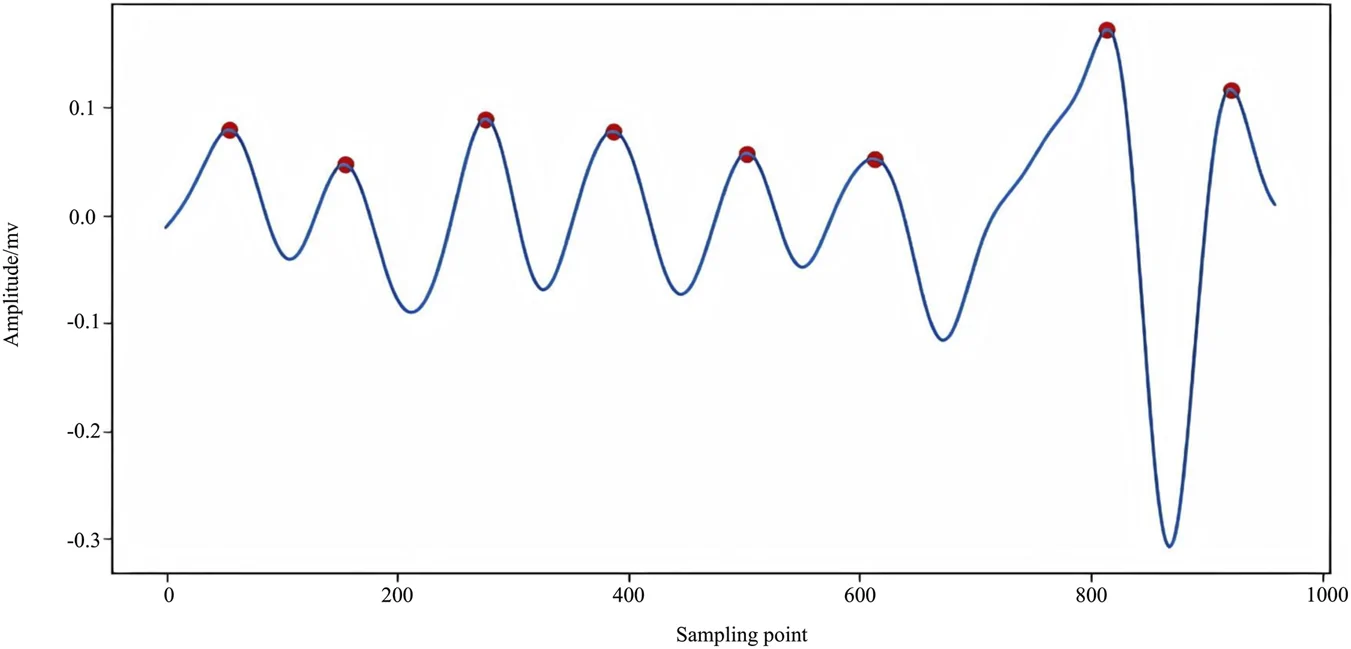
Detected respiratory peaks.

Valleys were detected using the same procedure applied to the inverted respiratory signal. The final detected respiratory peaks are illustrated in Figure 4.

### 2.4 Time-series construction and feature extraction

After peak detection, the RR intervals and respiratory peak intervals were first extracted from the detected R-peaks and respiratory peaks, respectively. To reduce the influence of measurement errors and abnormal beats, outlier removal was performed on the RR intervals. Specifically, intervals exceeding five standard deviations from the mean were considered abnormal and excluded.

After removing outliers, both RR intervals and respiratory peak intervals were resampled into uniformly sampled time series using linear interpolation at a sampling rate of 1 Hz. This step ensures temporal alignment and enables subsequent time-series modeling using fixed-length inputs.

Following interpolation, the time series were segmented using a sliding window approach. Each segment had a duration of 210 s, with a step size of 30 s between consecutive windows. The relatively long window length allows the model to capture sufficient temporal context, while the small step size increases the number of training samples and preserves temporal continuity.

For each 210 s window, the sleep stage label was assigned based on the annotation of the central 30 s epoch. This labeling strategy ensures consistency with standard sleep staging protocols, which typically use 30 s epochs as the basic unit of annotation.

Within each window, instantaneous heart rate (IHR) and instantaneous breathing rate (IBR) were computed. IHR was derived from the RR intervals as Equation 8:

$$\text{IHR}\left(t\right)=\frac{60}{\text{RR}\left(t\right)}$$ (8)

where RR(t) denotes the RR interval at time t in seconds. Similarly, IBR was calculated from the respiratory peak intervals as Equation 9:

$$\text{IBR}\left(t\right)=\frac{60}{\text{Resp}\left(t\right)}$$ (9)

where Resp(t) represents the interval between consecutive respiratory peaks.

Finally, Z-score normalization was applied to both IHR and IBR to standardize the feature distributions (Equation 10):

$$x^{\prime }=\frac{x‐\mu }{\sigma }$$ (10)

where μ and σ denote the mean and standard deviation of the corresponding signal. This normalization improves numerical stability and facilitates model convergence during training.

## 3 Model architecture

### 3.1 Overall architecture

**FIGURE 5:**
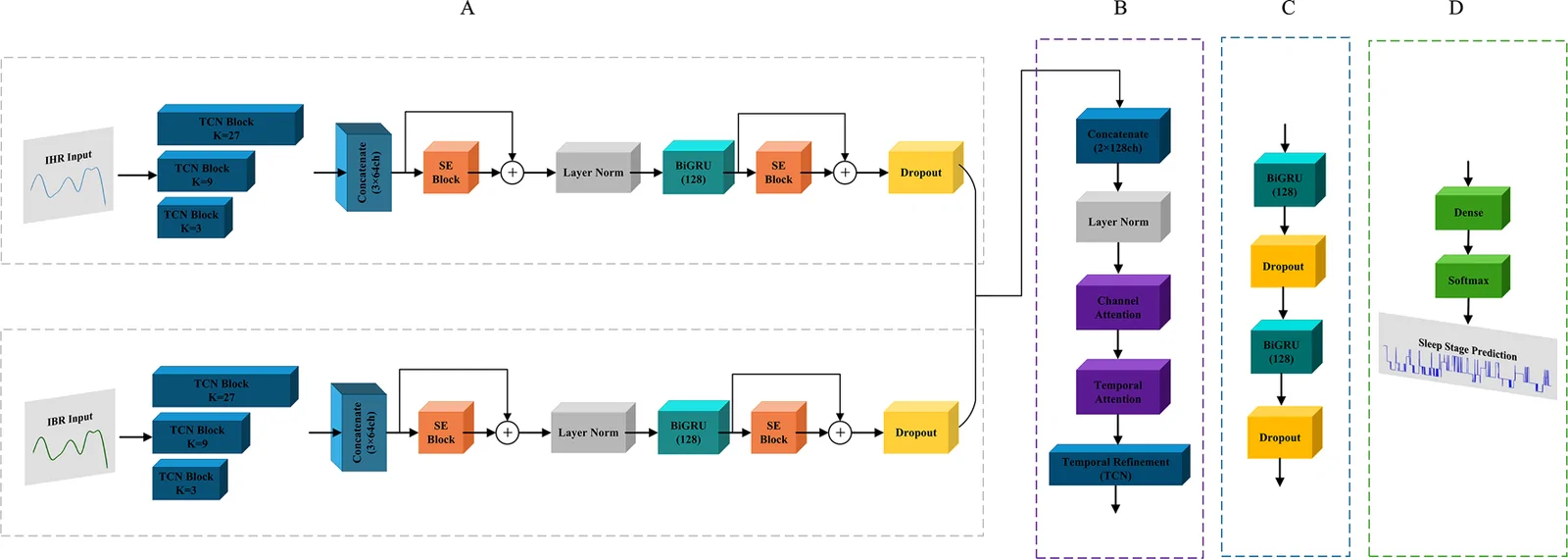
Overall architecture of the proposed TCN-BiGRU-SE model for sleep staging. **(A)** The instantaneous heart rate (IHR) and instantaneous breathing rate (IBR) signals are processed through two parallel branches. Each branch employs a multi-scale temporal convolutional network (TCN) with different kernel sizes (e.g., 3, 9, and 27) to capture temporal dependencies at multiple resolutions. Squeeze-and-excitation (SE) blocks are applied to enhance channel-wise feature representation. **(B)** The modality-specific features are concatenated and normalized using layer normalization. This step integrates information from both IHR and IBR modalities to form a unified feature representation. A hybrid attention mechanism is applied to the fused features, including both channel attention and temporal attention. This module adaptively emphasizes informative channels and time steps, improving the discriminative capability of the model. **(C)** The refined features are further processed by a temporal refinement module based on TCN to enhance temporal representation after fusion. Subsequently, stacked bidirectional gated recurrent units (BiGRU) are used to capture long-range temporal dependencies. Dropout is applied to prevent overfitting. **(D)** A fully connected layer with softmax activation outputs the final sleep stage probabilities.

To perform automatic sleep staging based on cardiorespiratory dynamics, we propose a multi-modal TCN-BiGRU-SE network using IHR and IBR signals. The overall architecture is illustrated in Figure 5. The model adopts a dual-branch structure, where IHR and IBR sequences are processed separately to extract modality-specific temporal features. The extracted features are subsequently fused and further modeled to capture cross-modal dependencies and long-range temporal dynamics, followed by a classification layer to generate sleep stage predictions.

This design is motivated by three considerations. First, IHR and IBR reflect complementary physiological processes during sleep, and independent feature extraction helps preserve modality-specific information. Second, sleep stages exhibit strong temporal continuity, requiring sequential modeling beyond local feature extraction. Third, different feature channels contribute unequally to classification, and channel-wise recalibration can enhance discriminative representations.

### 3.2 Structure of the modality-specific multi-scale feature extraction branch

The modality-specific branch is designed to extract temporal patterns from IHR and IBR signals separately before multi-modal fusion. As shown in Figure 5, the model adopts a dual-branch architecture, in which the IHR and IBR sequences are fed into two parallel feature extraction pathways with identical structures. This design allows the model to preserve the intrinsic characteristics of each physiological modality and avoid premature interference between heterogeneous signals.

For each modality, a multi-scale temporal convolutional network (TCN) is employed as the front-end feature extractor. Specifically, three parallel convolutional branches with kernel sizes of 3, 9, and 27 are used to capture temporal dependencies at different receptive-field scales. Since the IHR and IBR sequences are represented as uniformly sampled time series, these kernel sizes correspond to short-, medium-, and relatively long-range temporal contexts. The small kernel size of 3 focuses on short-term local fluctuations in heart rate and breathing rate, while the medium kernel size of 9 captures more stable rhythm changes over a wider temporal range. The larger kernel size of 27 provides broader contextual information and can cover slower cardiorespiratory variations across multiple breathing cycles, which are relevant to sleep-stage transitions. By combining features learned from different kernel sizes, the branch can simultaneously characterize rapid transitions and relatively slow variations in cardiorespiratory dynamics, which is beneficial for distinguishing sleep stages with different temporal patterns.

Each convolutional branch uses causal and dilated convolutions. The causal structure ensures that the output at the current time step depends only on the current and previous observations, thereby avoiding future information leakage during sequence modeling. Dilated convolution enlarges the receptive field without substantially increasing the number of parameters, enabling the network to model both short-range and long-range temporal dependencies efficiently. To improve gradient propagation and preserve the original signal information, residual connections are introduced within the TCN module. Through residual learning, the branch can extract deeper temporal representations while alleviating the degradation problem in stacked convolutional layers.

After the multi-scale temporal features are obtained, a squeeze-and-excitation (SE) block is applied to each modality-specific representation for intra-modal channel recalibration. This operation focuses on enhancing discriminative feature channels within each modality independently, without introducing cross-modal interactions at this stage. The SE mechanism first aggregates channel-wise global information through global average pooling and then learns adaptive channel weights using a lightweight gating structure. In this way, channels that contribute more to sleep-stage discrimination are emphasized, while less informative responses are suppressed. Since IHR and IBR contain different physiological cues, applying SE independently in each branch enables the network to enhance modality-relevant feature dimensions before feature fusion. In the SE block, the reduction ratio was set to 16. Given an input feature map with C channels, global average pooling was first applied along the temporal dimension to obtain a channel descriptor. The descriptor was then passed through two fully connected layers, where the first layer reduced the channel dimension from C to C/16 with ReLU activation, and the second layer restored the dimension from C/16 to C with sigmoid activation. The learned channel weights were then multiplied with the original feature map to recalibrate channel-wise feature responses. In this study, the modality-specific SE blocks were applied after the multi-scale TCN feature extraction of the IHR and IBR branches, and another SE block was applied after feature fusion to enhance informative fused representations.

Overall, the modality-specific multi-scale feature extraction branch aims to generate discriminative representations for each input modality by combining multi-scale temporal convolution and channel-wise recalibration. The extracted features provide a strong basis for subsequent multi-modal fusion and attention refinement.

### 3.3 Structure of the multi-modal fusion and attention refinement module

After modality-specific feature extraction, the high-level representations from the IHR and IBR branches are concatenated along the feature dimension to form a unified multi-modal representation, as illustrated in Figure 5. Compared with direct early fusion at the input level, this feature-level fusion strategy enables the model to integrate complementary information after each modality has been sufficiently encoded, thereby improving the representation capacity of the fused features.

Following concatenation, layer normalization is applied to the fused feature map to stabilize feature distribution across channels and time steps. This operation helps reduce internal covariate shift and facilitates subsequent optimization. On top of the normalized fused representation, a hybrid attention mechanism is introduced to refine the fused multi-modal representation, capturing both inter-channel dependencies and temporal dynamics across modalities. Unlike the SE blocks applied in the modality-specific branches, which perform intra-modal channel recalibration, the attention mechanism in this stage operates on the fused representation and is designed to model inter-modal interactions as well as temporal dependencies. The channel attention module reweights different feature channels according to their relative importance in the fused representation. Because the concatenated features contain information from different temporal scales and different physiological modalities, not all channels contribute equally to classification. Channel attention adaptively highlights the most informative feature dimensions and suppresses redundant or noisy components. In parallel, the temporal attention module assigns different weights to different time steps, enabling the network to focus on temporally salient regions that are more relevant to sleep-stage transitions. The combination of these two attention mechanisms allows the model to refine the fused features in both the feature-dimension and temporal-dimension spaces, thereby enhancing discriminative representation learning.

As shown in Figure 5, the attention-refined multi-modal features are further processed by a temporal refinement module based on TCN. This module is introduced after fusion to strengthen temporal interaction modeling across modalities. Unlike the modality-specific TCN branches that mainly learn intra-modal temporal patterns, the post-fusion TCN focuses on refining the joint temporal structure of the integrated cardiorespiratory representation. This step helps the model capture more coherent multi-modal dynamics before recurrent sequence modeling.

The refined features are then fed into stacked bidirectional gated recurrent unit (BiGRU) layers. The BiGRU processes the sequence in both forward and backward directions, which enables the model to learn contextual dependencies from preceding and succeeding time steps simultaneously. This bidirectional modeling is particularly suitable for sleep staging, where the state of a given epoch is often influenced by its surrounding temporal context. By stacking multiple BiGRU layers, the network can learn increasingly abstract sequential representations. Dropout is applied after recurrent processing to reduce overfitting and improve generalization performance.

Finally, as shown in Figure 5, the output sequence representation is mapped to the sleep-stage label space through a fully connected layer, and the posterior probabilities of different sleep stages are obtained using a softmax activation function. Through multi-modal fusion, hybrid attention refinement, post-fusion temporal enhancement, and bidirectional sequential modeling, the proposed module effectively integrates IHR and IBR information and improves the overall sleep staging performance.

In this way, the proposed framework forms a hierarchical attention strategy, where intra-modal feature enhancement is first achieved via SE blocks, followed by inter-modal and temporal refinement through hybrid attention after feature fusion.

## 4 Result analysis and discussion

### 4.1 Evaluation metrics

To comprehensively evaluate the performance of the proposed model, both overall metrics and class-wise metrics for each sleep stage were employed. Specifically, Precision, Recall, and F1-score were used to assess the classification performance for each sleep stage, while overall Accuracy and Cohen’s Kappa coefficient were used to evaluate the model performance on the entire dataset. These metrics are commonly used in sleep stage classification studies to provide a comprehensive evaluation of model performance (Xu et al., 2025).

Precision measures the proportion of correctly predicted positive samples among all samples predicted as a given sleep stage (Equation 11):

$$\mathrm{Pr}\text{ecision}=\frac{\text{TP}}{\text{TP}+\text{FP}}$$ (11)

Recall represents the proportion of correctly predicted samples among all actual samples of a given sleep stage (Equation 12):

$$\text{Recall}=\frac{\text{TP}}{\text{TP}+\text{FN}}$$ (12)

The F1-score is the harmonic mean of Precision and Recall (Equation 13):

$$F1-\text{score}=2\cdot \frac{\mathrm{Pr}\text{ecision}\cdot Recall}{\mathrm{Pr}\text{ecision}+\text{Recall}}$$ (13)

Accuracy reflects the proportion of correctly classified samples over all samples (Equation 14):

$$\text{Accuracy}=\frac{\text{TP}+\text{TN}}{\text{TP}+\text{FP}+\text{TN}+\text{FN}}$$ (14)

where TP, TN, FP, and FN denote true positive, true negative, false positive, and false negative samples, respectively.

Cohen’s Kappa coefficient is used to measure the agreement between the predicted labels and the true labels while accounting for agreement occurring by chance (Equation 15):

$$\text{Kappa}=\frac{P_{o}‐P_{e}}{1‐P_{e}}$$ (15)

where $P_{o}$ represents the observed agreement (i.e., Accuracy), and $P_{e}$ denotes the expected agreement by chance. The value of Kappa ranges from −1 to 1, where a higher value indicates better agreement between predictions and ground truth.

### 4.2 Experimental details

The proposed model was evaluated on two publicly available sleep datasets. A total of 7,837 overnight recordings from the Sleep Heart Health Study (SHHS) dataset were selected for model development. To avoid data leakage and evaluate the model’s generalization ability to unseen subjects, the SHHS dataset was randomly divided at the subject level into training, validation, and test sets in a ratio of 8:1:1. All overnight recordings and sleep epochs from the same subject were assigned to only one subset, and no subject appeared in more than one of the training, validation, or test sets.

To further assess the generalization capability of the model under different data distributions, an external dataset, the Multi-Ethnic Study of Atherosclerosis (MESA), was introduced for independent testing. The MESA dataset consists of 1,970 overnight sleep recordings, which were not involved in the training process.

The model was implemented using the Keras deep learning framework and trained on a workstation equipped with an NVIDIA RTX 2080Ti GPU. During training, the batch size was set to 32. The categorical cross-entropy loss function was used to optimize the multi-class classification task. The Adam optimizer was adopted, with the initial learning rate set in the range of 1e-4 to 1e-3.

The maximum number of training epochs was set to 100. Early stopping was applied based on the validation loss, and training was terminated if the validation loss did not decrease for 10 consecutive epochs. In addition, a learning rate scheduling strategy was employed: if the validation loss did not improve for 5 epochs, the learning rate was reduced to 50% of its previous value. These strategies help stabilize the training process and prevent overfitting.

The main architectural and training parameters are summarized in Table 2.

**TABLE 2:** Main architectural and training parameters of the proposed model.

Parameter	Value
Input modalities	IHR and IBR
Window length	210 s
Sampling rate after interpolation	1 Hz
TCN kernel sizes	3, 9, 27
Number of TCN filters	64
SE reduction ratio	16
Dropout rate	0.4
Loss function	Categorical cross-entropy
Optimizer	Adam
Batch size	32
Maximum epochs	100
Initial learning rate	1e-4 to 1e-3 (factor 0.5 if validation loss did not improve for 5 epochs)
Hardware	NVIDIA RTX 2080Ti GPU

### 4.3 Results

#### 4.3.1 Overall staging performance

The experimental results on the SHHS and MESA datasets are presented in Tables 3, 4, respectively. The proposed model was evaluated for sleep stage classification using IHR and IBR as input features. Overall, the model demonstrates strong performance in Wake and Light stages, while achieving moderate performance in REM and Deep stages, indicating stable classification capability across different sleep states.

**TABLE 3:** Sleep stage classification performance on the SHHS dataset.

Stage	Wake	REM	Light	Deep	Recall (%)	Precision (%)	F1-score (%)
Wake	237,825	4,314	24,015	430	89.21	90.71	89.95
REM	2,477	96,198	12,979	229	85.98	82.58	84.24
Light	20,972	15,196	287,863	29,735	81.37	79.66	80.51
Deep	918	787	36,487	63,621	62.49	67.67	64.98
Overall accuracy: 82.19% kappa: 0.74							

**TABLE 4:** Sleep stage classification performance on the MESA dataset.

Stage	Wake	REM	Light	Deep	Recall (%)	Precision (%)	F1-score (%)
Wake	917,459	30,484	104,625	3,520	86.88	90.28	88.54
REM	11,113	212,294	34,795	1,057	81.90	72.03	76.66
Light	84,153	50,507	778,966	105,074	76.46	79.96	78.18
Deep	3,472	1,264	55,786	84,618	58.30	43.56	49.89
Overall accuracy: 80.44% Kappa: 0.70							

On the SHHS dataset, the proposed model achieved an overall accuracy of 82.19% and a Cohen’s Kappa coefficient of 0.74, indicating substantial agreement between predicted labels and ground truth. Among all sleep stages, the Wake stage achieved the highest performance, with an F1-score of 89.95%. This can be attributed to the relatively distinct physiological characteristics of the wake state, where heart rate and breathing patterns differ significantly from sleep stages, making it easier for the model to discriminate.

The Light stage also showed strong performance, with an F1-score of 80.51%. This suggests that the proposed multi-scale TCN is effective in capturing temporal variations in IHR and IBR signals associated with light sleep. However, the performance in REM and Deep stages is relatively lower. This may be due to the similarity of physiological patterns between these stages and other sleep states. In particular, Deep sleep is characterized by relatively stable and low-variability cardiorespiratory signals, which reduce feature separability. In contrast, REM sleep exhibits more irregular patterns, which may overlap with Wake or Light stages, increasing classification difficulty.

On the MESA dataset, the model achieved an overall accuracy of 80.44% and a Kappa coefficient of 0.70, demonstrating good generalization performance across datasets with different distributions. The Wake stage again achieved the best performance, with an F1-score of 88.54%, consistent with the results observed on the SHHS dataset. The Light stage maintained relatively stable performance (F1-score of 78.18%), further confirming the robustness of the model in identifying dominant sleep patterns.

Although the performance in REM and Deep stages remains lower, the model still captures partial discriminative features, indicating potential for further improvement. The consistent performance across SHHS and MESA datasets suggests that the proposed multi-modal framework effectively leverages complementary information from IHR and IBR signals, enhancing its ability to generalize across different populations.

As shown in Table 1, the sleep-stage distributions were imbalanced in both datasets. Wake and Light accounted for the majority of samples, whereas REM and Deep were relatively underrepresented, especially Deep sleep in the MESA dataset. This imbalance may partly contribute to the lower F1-scores observed for REM and Deep stages, because the model receives fewer training examples for these minority classes and the physiological patterns of these stages are less separable from adjacent sleep stages.

#### 4.3.2 Sleep stage temporal sequence analysis

The purpose of automatic sleep staging is to provide an efficient and reliable tool for clinical sleep assessment. Temporal sequence visualization offers an intuitive way to evaluate the consistency between predicted sleep stages and ground truth labels over time.

**FIGURE 6:**
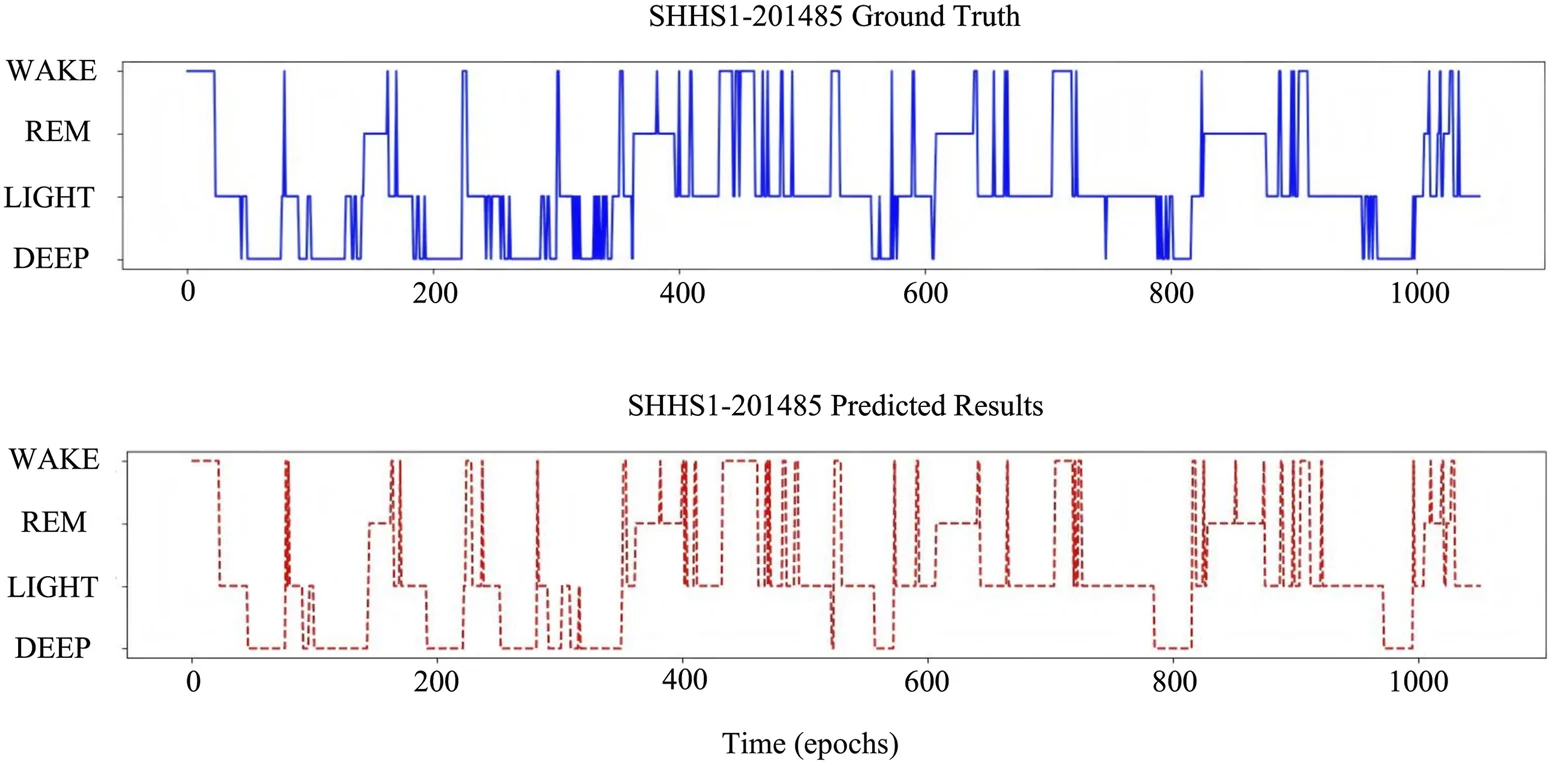
Comparison of predicted and ground truth sleep stage sequences (SHHS dataset).

**FIGURE 7:**
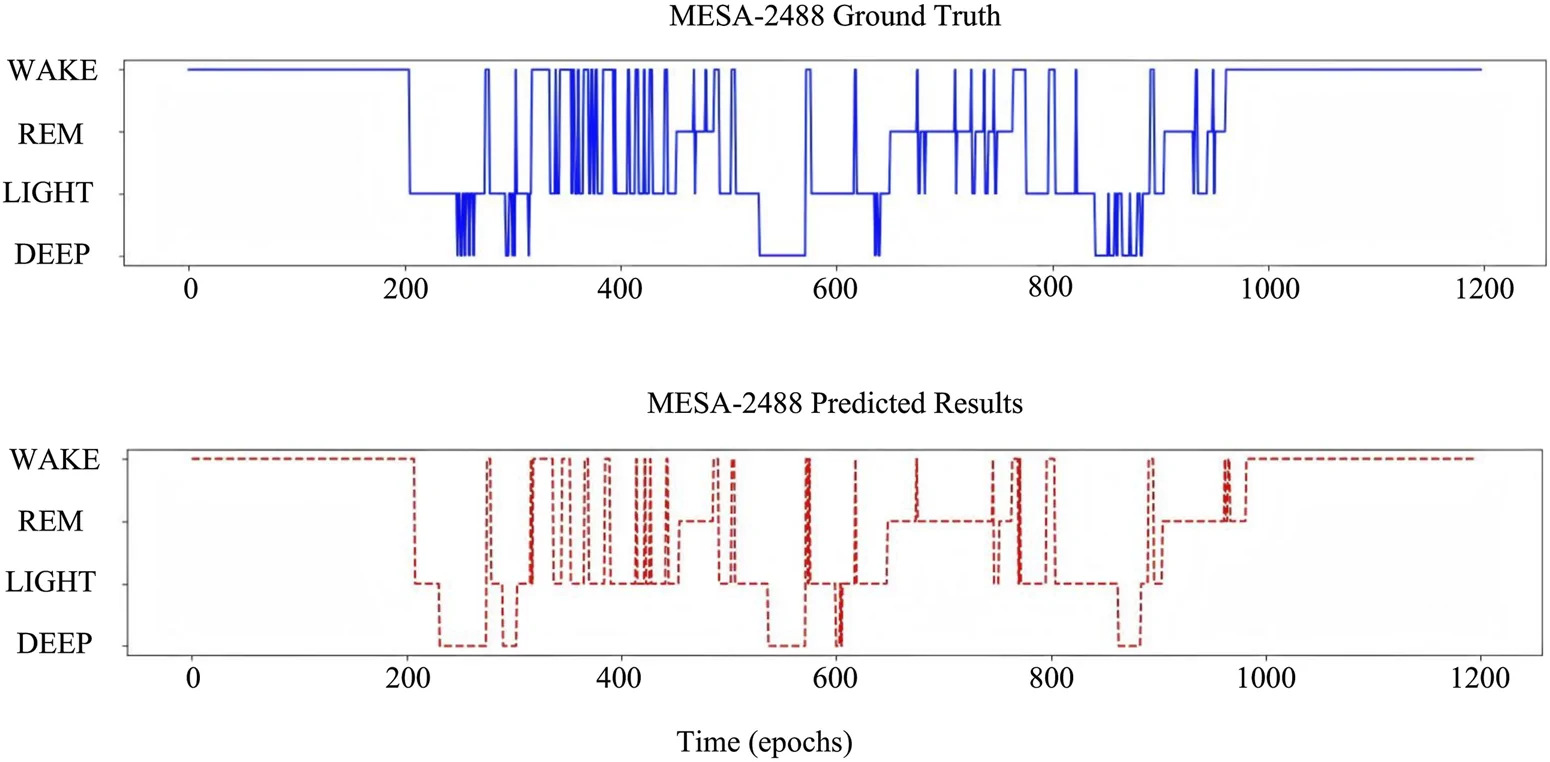
Comparison of predicted and ground truth sleep stage sequences (MESA dataset).

To assess the temporal performance of the proposed model, the predicted sleep stage sequences were compared with manually annotated labels for representative subjects. As illustrated in Figures 6, 7, the model can effectively capture the overall transition patterns of sleep stages throughout the night.

In particular, the predicted sequences show high consistency with the ground truth in the Wake and Light stages, where stage boundaries are relatively clear. The model successfully tracks long-duration stable segments and accurately identifies transitions between major sleep states. This indicates that the proposed framework can effectively model temporal dependencies in cardiorespiratory signals.

However, some discrepancies can be observed in REM and Deep stages. These mismatches are mainly concentrated around transition regions, where sleep stages change rapidly and physiological patterns become less distinguishable. This behavior is consistent with the quantitative results, where lower performance was observed for these stages.

Overall, the temporal sequence results demonstrate that the proposed model not only achieves good classification performance at the epoch level but also maintains temporal coherence in sleep stage prediction. This is particularly important for practical applications, as clinically meaningful sleep analysis requires consistent stage transitions over time rather than isolated predictions.

#### 4.3.3 Comparison with existing methods

To further evaluate the effectiveness of the proposed model, a comparison with several representative sleep staging methods based on ECG and respiratory signals was conducted. The comparison results on public datasets are summarized in Table 5.

**TABLE 5:** Comparison between our proposed method and other approaches.

References	Input	Method	Classes	Dataset	Accuracy (%)	Kappa
Sridhar et al. (2020)	IHR	CNN	4	SHHS	77.3%	0.66
​	​	​	4	MESA	80%	0.69
Mitsukura et al. (2020)	HR	RNN	4	Private	72%	-
Bakker et al. (2021)	IHR,Resp	CReSS	4	SHHS	76.7%	0.64
​	​	​	4	MESA	79.8%	0.68
Tang et al. (2022)	ECG	TL	4	SHHS1	78.7%	0.75
​	​	​	4	SHHS2	74.8%	0.67
Proposed method	IHR,IBR	TCN-BiGRU-SE	4	SHHS	82.19%	0.74
​	​	​	4	MESA	80.44%	0.70

As shown in Table 5, the proposed TCN-BiGRU-SE model achieves competitive performance compared with existing methods. On the SHHS dataset, the proposed model achieves an accuracy of 82.19% and a Kappa coefficient of 0.74, outperforming several traditional and deep learning-based approaches, such as CNN-based and RNN-based methods. On the MESA dataset, the model also demonstrates strong generalization ability, achieving an accuracy of 80.44% and a Kappa of 0.70.

Compared with methods that rely on single-modality inputs, the proposed model benefits from the integration of IHR and IBR signals, which provide complementary physiological information related to cardiovascular and respiratory dynamics. This multi-modal design improves the model’s ability to distinguish between different sleep stages.

Furthermore, the use of a multi-scale TCN enables the model to capture temporal features at different resolutions, allowing it to better represent both short-term fluctuations and long-term dependencies in physiological signals. This contributes to improved performance, particularly in stages with complex temporal patterns.

In addition, the incorporation of attention mechanisms enhances feature representation by adaptively focusing on informative channels and time steps. The subsequent BiGRU layers further model bidirectional temporal dependencies, which helps improve sequence-level consistency in sleep stage prediction.

The proposed model should be distinguished from previous multimodal sleep-staging methods and single-attention architectures. Many existing studies rely on PSG signals, such as EEG, EOG, and EMG, or use a single temporal modeling strategy. In contrast, our method focuses on IHR and IBR signals and combines multi-scale temporal convolution, bidirectional recurrent modeling, and SE-based feature recalibration in a unified framework. This design allows the model to learn complementary information from cardiac and respiratory dynamics while adaptively emphasizing informative feature channels after modality-specific extraction and multimodal fusion.

Overall, the comparison results demonstrate that the proposed model effectively combines multi-scale temporal modeling, multi-modal feature fusion, and attention-based refinement, leading to improved performance and generalization across different datasets.

However, compared with some methods on specific datasets, the proposed model shows slightly lower performance in certain stages, which may be due to differences in dataset characteristics and class imbalance.

#### 4.3.4 Ablation study

To investigate the contribution of different modules to the overall performance, a series of ablation experiments were conducted on the SHHS dataset. Six model variants were designed to evaluate the individual and combined effects of the TCN, BiGRU, and SE modules.

Specifically, the following configurations were considered: (1) TCN-only model, (2) BiGRU-only model, (3) TCN-BiGRU model without SE, (4) TCN-SE model without BiGRU, (5) BiGRU-SE model without TCN, and (6) the full TCN-BiGRU-SE model.

The experimental results are summarized in Table 6. As shown in the table, different module combinations have distinct impacts on sleep staging performance.

**TABLE 6:** Ablation study results on the SHHS dataset.

Methods	Accuracy (%)	Recall (%)	Precision (%)	F1-score (%)	Kappa
TCN	76.59	71.40	74.75	72.43	0.66
BiGRU	77.82	76.30	72.30	73.52	0.67
TCN-BiGRU	79.90	74.39	78.99	75.98	0.70
TCN-SE	78.42	72.86	78.17	75.00	0.68
BiGRU-SE	78.62	75.61	76.63	76.09	0.69
TCN-BiGRU-SE	82.19	79.76	80.16	79.92	0.74

The TCN-only model achieves relatively lower performance, indicating that although TCN is effective in capturing local temporal features, it lacks the capability to model long-term dependencies. In contrast, the BiGRU-only model shows improved performance, demonstrating its advantage in modeling sequential dependencies in time-series data.

When combining TCN and BiGRU, the performance is further improved, suggesting that the multi-scale temporal features extracted by TCN can enhance the sequence modeling capability of BiGRU. However, without the SE module, the improvement remains limited.

The introduction of the SE module leads to consistent performance gains. Both TCN-SE and BiGRU-SE models outperform their counterparts without SE, indicating that channel-wise feature recalibration helps emphasize informative features and suppress irrelevant information.

Notably, the BiGRU-SE model achieves better performance than the TCN-SE model, suggesting that the SE mechanism is particularly effective when applied to sequence modeling features. This highlights the importance of adaptive feature weighting in temporal modeling tasks.

The full TCN-BiGRU-SE model achieves the best performance across all evaluation metrics, demonstrating that the combination of multi-scale feature extraction, temporal dependency modeling, and channel attention provides complementary benefits. These results confirm that TCN, BiGRU, and SE modules collaboratively contribute to improved sleep stage classification performance.

### 4.4 Computational complexity and deployment considerations

The proposed model contains approximately 7.0 million trainable parameters. Compared with PSG-based sleep staging models that require multiple high-dimensional signals such as EEG, EOG, and EMG, the proposed framework uses only two low-dimensional cardiorespiratory time series, IHR and IBR, which reduces the input complexity and makes the method more suitable for low-burden sleep monitoring. After ECG and respiratory peak detection, the model performs inference on uniformly sampled IHR and IBR sequences with a 210 s temporal context and a 30 s prediction step. Therefore, the model can be applied to overnight sleep recordings in an offline or batch-processing manner.

For wearable deployment, the main computational requirements include reliable R-peak and respiratory peak detection, interpolation of IHR and IBR sequences, and neural network inference using the trained TCN-BiGRU-SE model. Since the current model uses BiGRU layers to capture bidirectional temporal context, it is more suitable for retrospective overnight sleep analysis than strict real-time sleep staging. For future wearable deployment, lightweight optimization strategies such as model pruning and quantization may help reduce memory and computational costs on resource-constrained devices (Lin et al., 2023). Future work will also explore causal or unidirectional temporal modeling to improve real-time applicability.

## 5 Conclusion

In this study, an automatic sleep staging method based on IHR and IBR was proposed. A TCN-BiGRU-SE model was constructed to perform end-to-end sleep stage classification. By integrating the local feature extraction capability of temporal convolutional networks (TCN), the sequence modeling strength of bidirectional gated recurrent units (BiGRU), and the channel-wise feature recalibration of squeeze-and-excitation (SE) modules, the proposed framework effectively improves the representation of multi-modal cardiorespiratory signals.

The experimental results on the SHHS and MESA datasets demonstrate that the proposed model achieves competitive performance in sleep stage classification. In particular, the model shows strong performance in Wake and Light stages, indicating its effectiveness in capturing dominant temporal patterns. The use of multi-scale temporal convolution and multi-modal fusion reduces the dependence on handcrafted features and enhances the model’s ability to learn discriminative representations directly from raw physiological signals.

However, the model still exhibits relatively lower performance in REM and Deep stages. This limitation is mainly attributed to the similarity and instability of physiological patterns in these stages. Deep sleep is characterized by relatively stable and low-variability cardiorespiratory signals, which leads to reduced feature separability. In contrast, REM sleep presents more irregular patterns, which may overlap with Wake and Light stages in terms of IHR and IBR characteristics, increasing classification difficulty.

Future work will focus on improving the discrimination of challenging sleep stages by incorporating additional physiological signals and more advanced temporal modeling strategies. Furthermore, transfer learning techniques will be explored to extend the proposed framework to other sensing modalities, such as ballistocardiography signals, respiratory belt signals, nasal airflow signals, and radar-based cardiopulmonary measurements, thereby enhancing the applicability of the model in real-world sleep monitoring scenarios.

## Data Availability

Publicly available datasets were analyzed in this study. This data can be found here: https://sleepdata.org/datasets.
